# Quercetin ameliorates gestational diabetes mellitus-induced placental dysfunction by modulating the AGE-RAGE-associated oxidative and inflammatory signaling network

**DOI:** 10.3389/fphar.2026.1928244

**Published:** 2026-10-22

**Authors:** Xinying Liu, Xingyi Qi, Zewei Liang, Chengcheng Jin, Xiuli Li, Xia Xu, Jianying Yan

**Affiliations:** 1 College of Clinical Medicine for Obstetrics and Gynecology and Pediatrics, Fujian Medical University Fujian Maternity and Child Health Hospital, Fuzhou, Fujian, China; 2 Fujian Clinical Research Center for Maternal-Fetal Medicine, Fuzhou, Fujian, China; 3 Laboratory of Maternal-Fetal Medicine, Fujian Maternity and Child Health Hospital, Fuzhou, Fujian, China; 4 National Key Obstetric Clinical Specialty Construction Institution, Fuzhou, Fujian, China

**Keywords:** AGE-RAGE, gestational diabetes mellitus, inflammation, quercetin, reactive oxygen species

## Abstract

**Background:**

Gestational diabetes mellitus (GDM) induces placental structural and functional disturbances associated with dysregulated AGE-RAGE/ROS signaling and inflammatory responses. Existing flavonoid-related studies merely observed changes in pathway protein levels, lacking direct experimental proof of molecular binding between candidate compounds and core receptors. This study aimed to investigate the potential involvement of RAGE in the protective effects of quercetin and to characterize the associated AGE-RAGE/ROS/NF-κB-related signaling changes in GDM placental injury.

**Methods:**

Network pharmacology was used to screen quercetin’s therapeutic targets for GDM. Molecular docking was performed using the human RAGE structure to predict the potential interaction between quercetin and RAGE, and molecular dynamics simulation was conducted to evaluate the stability of the predicted complex. A combined streptozotocin-high-fat diet GDM rat model and 30 mM high-glucose HTR-8/SVneo trophoblast injury model were constructed. FPS-ZM1 was used as a pharmacological tool to perturb RAGE-associated signaling *in vitro*. ROS, inflammatory cytokines, and key pathway gene and protein expression were examined to characterize the associated signaling changes.

**Results:**

Network pharmacology identified SERPINE1 (a critical regulator of placental vascular remodeling) as the core hub gene, with the AGE-RAGE pathway showing the highest enrichment score. Molecular docking and molecular dynamics simulations predicted a dynamically stable complex formed between quercetin and the ligand-binding domain of RAGE. GDM induction was associated with increased AGE accumulation, RAGE expression, ROS production, phosphorylated NF-κB p65, and SERPINE1 expression, together with an increase in Nrf2 expression, whereas quercetin treatment attenuated these alterations. Quercetin intervention also improved glucose tolerance and placental pathological changes.

**Conclusion:**

Quercetin alleviates GDM-associated placental dysfunction by improving metabolic abnormalities, oxidative imbalance, and inflammatory responses. Computational analysis suggests a potential interaction between quercetin and RAGE. These findings provide evidence that quercetin may regulate the AGE-RAGE-associated oxidative and inflammatory signaling network and improve placental homeostasis under diabetic conditions.

## Graphical Abstract

**Figure:**
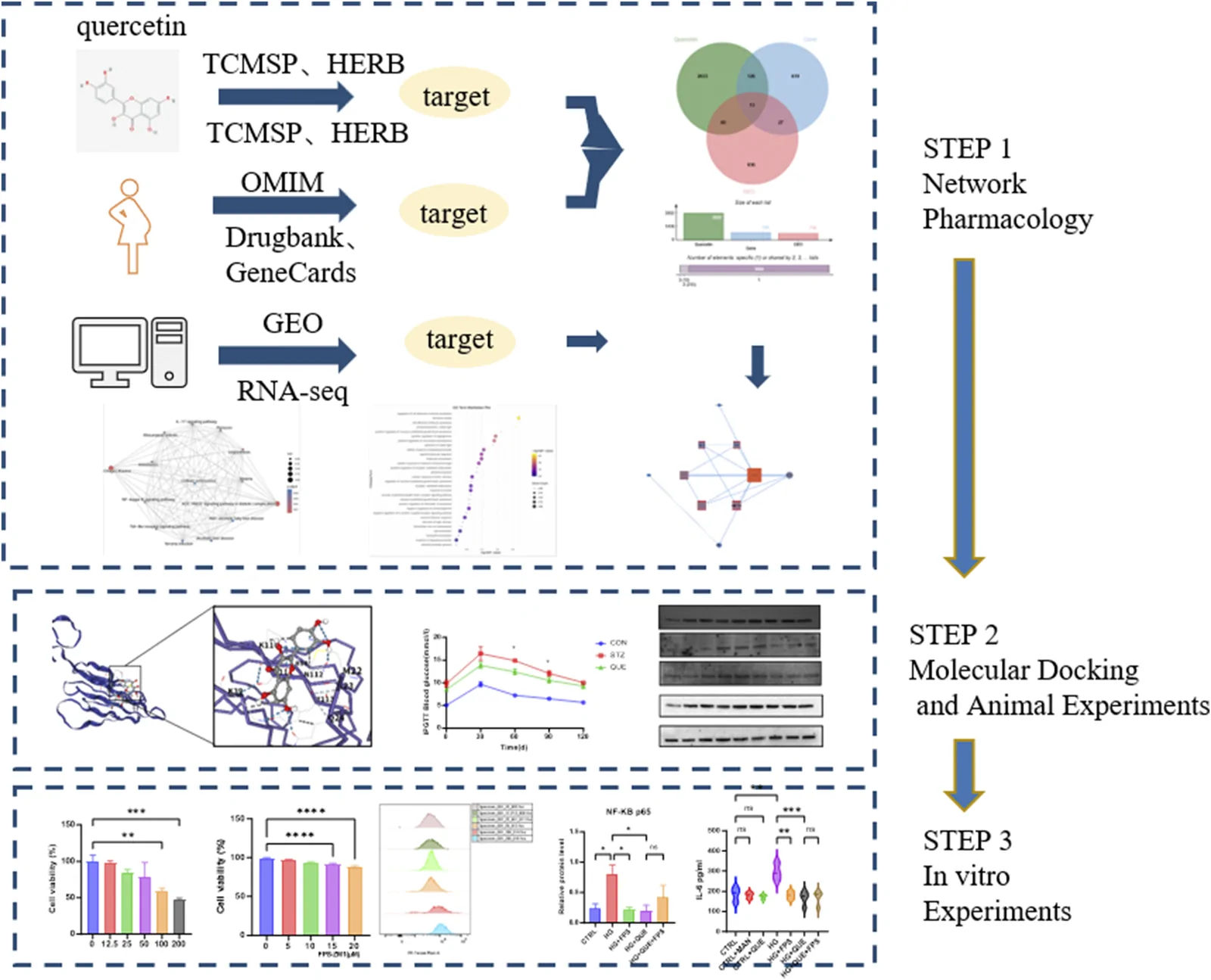

## 1 Introduction

Gestational diabetes mellitus (GDM) is a common pregnancy-associated metabolic disorder characterized by impaired glucose tolerance and insulin resistance that develops during pregnancy. GDM affects a considerable proportion of pregnancies worldwide and is associated with increased risks of adverse maternal and fetal outcomes, including hypertensive disorders, fetal overgrowth, and long-term metabolic disorders in offspring (McIntyre et al., 2019; Plows et al., 2018). Although maternal hyperglycemia represents the primary metabolic abnormality of GDM, increasing evidence indicates that the placenta is not merely a passive target of metabolic disturbance but an active regulator of maternal–fetal metabolic communication. Alterations in placental metabolism, inflammatory status, and vascular function contribute substantially to the development and progression of GDM-associated complications (Desoye and Hauguel-de Mouzon, 2007).

Under physiological conditions, the placenta maintains a precisely regulated balance between oxidative metabolism, antioxidant defense, and inflammatory responses to support fetal growth and maternal adaptation. However, persistent exposure to hyperglycemic conditions disrupts this balance, resulting in excessive reactive oxygen species (ROS) generation, mitochondrial dysfunction, and inflammatory activation (Lappas et al., 2011). The placenta exhibits high metabolic activity and abundant mitochondrial content, making it particularly vulnerable to oxidative injury under diabetic conditions. Previous studies have demonstrated increased oxidative stress markers and impaired antioxidant capacity in placental tissues from women with GDM, suggesting that redox imbalance is a fundamental mechanism contributing to placental dysfunction (Coughlan et al., 2004; Brownlee, 2001).

Among the molecular mechanisms linking hyperglycemia to tissue injury, advanced glycation end products (AGEs) and their receptor RAGE have attracted considerable attention. AGEs are generated through nonenzymatic glycation reactions during chronic hyperglycemic exposure and accumulate in various diabetic complications. Binding of AGEs to RAGE activates intracellular signaling cascades that promote oxidative stress, inflammation, and cellular dysfunction (Brownlee, 2005). The AGE-RAGE axis has been recognized as a major contributor to diabetic vascular injury and metabolic complications through amplification of ROS production and activation of inflammatory pathways, particularly nuclear factor-κB (NF-κB)-dependent signaling (Bierhaus et al., 2005; Morgan and Liu, 2011; Mittal et al., 2014). Therefore, dysregulation of AGE-RAGE-associated signaling may represent an important molecular mechanism underlying GDM-associated placental injury.

Oxidative stress is closely controlled by endogenous antioxidant defense systems. Nuclear factor erythroid 2-related factor 2 (Nrf2) is a central transcriptional regulator responsible for maintaining cellular redox homeostasis through regulation of antioxidant and cytoprotective genes. Activation of the Keap1-Nrf2 pathway represents an important adaptive response against oxidative challenges; however, alterations in Nrf2 signaling may vary depending on cellular context, disease stage, and the intensity of oxidative stress (Kensler et al., 2007; Ma, 2013). Therefore, understanding the interaction between oxidative injury and antioxidant regulatory mechanisms is essential for elucidating the complex biological responses occurring during GDM-associated placental dysfunction.

In addition to oxidative and inflammatory abnormalities, metabolic stress may influence extracellular matrix remodeling and vascular regulation through molecules involved in tissue homeostasis. SERPINE1 encodes plasminogen activator inhibitor-1 (PAI-1), a multifunctional protein involved in fibrinolysis, extracellular matrix remodeling, angiogenesis, and inflammatory regulation. Increased SERPINE1 expression has been associated with insulin resistance, metabolic syndrome, and vascular complications, suggesting its potential involvement in diabetes-related tissue injury (Alessi and Juhan-Vague, 2006). However, the role of SERPINE1 in GDM-associated placental dysfunction and its regulation by protective interventions remain incompletely understood.

Quercetin is a naturally occurring flavonoid widely distributed in fruits and vegetables and has attracted extensive attention because of its antioxidant, anti-inflammatory, and metabolic regulatory properties (Boots et al., 2008). Previous studies have demonstrated that quercetin can alleviate oxidative stress and inflammatory injury in various diabetic models, suggesting its potential value in metabolic disease intervention (Eid and Haddad, 2017). However, whether quercetin protects against GDM-associated placental injury through regulation of upstream signaling networks remains unclear.

Network pharmacology and computational approaches provide powerful strategies for identifying potential targets and biological pathways of bioactive compounds. When combined with experimental validation, these approaches can facilitate systematic investigation of complex molecular mechanisms. Therefore, the present study integrated network pharmacology, molecular docking, molecular dynamics simulation, HFD/STZ-induced GDM animal experiments, and trophoblast cell validation to investigate whether quercetin ameliorates GDM-associated placental injury through modulation of AGE-RAGE-associated oxidative and inflammatory signaling pathways.

## 2 Materials and methods

### 2.1 Network pharmacology and bioinformatics analysis

#### 2.1.1 Prediction of potential targets of quercetin

Quercetin targets were screened from 5 databases, with their original websites and references as follows: ChEMBL (https://www.ebi.ac.uk/chembl/), Herb (http://herb.ac.cn/), SwissTargetPrediction (http://www.swisstargetprediction.ch/), SEA (http://sea.bkslab.org/), TSCMP (http://www.tscmp.com/). Targets with confidence score >0.7 were retained, and duplicate targets were removed using Excel to obtain unique quercetin targets. Quercetin’s basic information was retrieved from PubChem (https://pubchem.ncbi.nlm.nih.gov/).

#### 2.1.2 Collection of GDM-related genes

Three authoritative disease databases, Drugbank, GeneCards and OMIM, were used to collect genes associated with gestational diabetes mellitus. Genes with a relevance score >10 were included to ensure high correlation with disease pathogenesis. After deduplication, 831 valid GDM-related genes were acquired.

#### 2.1.3 Screening of differentially expressed genes from GEO dataset

The human placental transcriptome dataset GSE263483 was downloaded from the Gene Expression Omnibus (GEO, https://www.ncbi.nlm.nih.gov/geo/). R software (version 4.3.0) combined with the limma package was used for differential gene analysis. Genes satisfying the criteria of |log_2_FC| ≥2 and P < 0.05 were defined as differentially expressed genes (DEGs). Finally, 716 DEGs were screened out.

#### 2.1.4 Hierarchical venn intersection analysis

A two-step hierarchical intersection strategy was applied. Firstly, GDM-related genes were intersected with DEGs from the GEO dataset to obtain key genes with aberrant expression in GDM placenta. Secondly, the above key genes were further intersected with quercetin target genes to screen core genes regulated by quercetin in the treatment of GDM.

##### 2.1.4.1 PPI network and enrichment analysis

The core genes were imported into the STRING database (https://string-db.org/) to construct a protein-protein interaction (PPI) network, with the confidence threshold set at 0.7. Cytoscape 3.9.1 software was used for network visualization and hub gene identification. Gene Ontology (GO) and Kyoto Encyclopedia of Genes and Genomes (KEGG) enrichment analyses were performed via the clusterProfiler package in R software. *P* < 0.05 was considered statistically significant.

#### 2.1.5 Molecular docking

The potential interaction between quercetin and RAGE was predicted using the CB-Dock2 web server (http://cadd.labshare.cn/cb-dock2/), an automated cavity detection-guided blind docking platform based on the AutoDock Vina algorithm. The three-dimensional crystal structure of human RAGE (PDB ID: 3CJJ) was obtained from the RCSB Protein Data Bank, and the chemical structure of quercetin was obtained from the PubChem database. The prepared protein and ligand structures were submitted to the CB-Dock2 platform for blind docking analysis. The optimal docking conformation was selected according to the Vina docking score, and the predicted interaction pattern between quercetin and RAGE was analyzed based on the involved amino acid residues and intermolecular interactions.

#### 2.1.6 Molecular dynamics

The quercetin-RAGE complex generated from CB-Dock2 docking analysis was further subjected to molecular dynamics simulation to evaluate the stability of the predicted complex. The structural stability and dynamic characteristics of the complex were assessed by calculating root mean square deviation (RMSD), root mean square fluctuation (RMSF), radius of gyration (Rg), solvent accessible surface area (SASA), and hydrogen bond interactions during the simulation period.

### 2.2 Animals and experimental design

Specific pathogen-free (SPF) Sprague-Dawley rats (body weight: 200–220 g) were purchased from Suzhou biotechnology Co., Ltd. All rats were housed under standard laboratory conditions, with free access to standard chow and sterile drinking water. After 7 days of adaptive feeding, male and female rats were mated at a ratio of 1:1. The day when sperm was detected in vaginal smears was defined as gestational day 0 (GD0).

To evaluate both the effects of quercetin under physiological pregnancy conditions and its protective effects against GDM-associated injury, pregnant rats were randomly assigned into four groups: control group (CON), control plus quercetin group (CON + QUE), GDM model group (GDM), and GDM plus quercetin (100 mg/kg) treatment group (QUE). Each group consisted of 6–8 biological replicates, with each replicate derived from an independent maternal dam to minimize litter-related bias. The GDM model was induced by high-fat-diet feeding starting at 4 weeks of age for 4 consecutive weeks. Upon reaching 8 weeks of age, rats were mated. After successful mating, streptozotocin (STZ, MedChemExpress, 35 mg/kg) was intraperitoneally injected on gestational day 5 (GD5). Rats with random blood glucose ≥7.6 mmol/L were regarded as successfully modeled.

Quercetin (≥98% purity, APEBIO, Cat. No. N1841) was suspended in 1% sodium carboxymethyl cellulose solution and administered by intragastric gavage once a day from GD0 to GD20. The CON + QUE group received quercetin administration under normoglycemic pregnancy conditions, whereas the QUE group received the same quercetin administration regimen under GDM conditions. The CON and GDM groups received an equal volume of vehicle solution. On GD20, all rats were anesthetized, and blood and placental tissues were collected for subsequent detection. All animal experiments were approved by the Animal Ethics Committee of Fujian Maternity and Child Health Hospital and performed in accordance with institutional ethical guidelines (Approval No. AEC-SFY-XM2026-139).

#### 2.2.1 Intraperitoneal glucose tolerance test (IPGTT) and insulin tolerance test (ITT)

Intraperitoneal glucose tolerance test (IPGTT) was conducted on gestational day 16. All pregnant rats were fasted for 12 h with free access to drinking water before detection. Fasting blood glucose was measured at 0 min, followed by intraperitoneal injection of 2 g/kg glucose solution. Blood glucose levels were determined at 30, 60, 90 and 120 min after administration. The blood glucose curve was drawn and the area under the curve (AUC) was calculated to evaluate glucose tolerance status and insulin resistance. ITT was performed on gestational day 18. Rats were fasted for 6 h and then injected intraperitoneally with recombinant human insulin (0.75 U/kg, Eli Lilly, United States). Blood glucose was measured at 0, 15, 30, 60, and 120 min post-injection. The AUC was calculated to assess systemic insulin sensitivity.

#### 2.2.2 ROS detection

The intracellular ROS level of HTR-8/SVneo cells was detected by flow cytometry with DCFH-DA fluorescent probe. The ROS content in rat placental tissue was determined using a fluorescence-based ROS detection assay kit (Youpin Biotechnology, China) according to the manufacturer’s instructions. The fluorescence intensity was measured using a fluorescence microplate reader, and ROS levels were expressed as relative fluorescence intensity.

#### 2.2.3 ELISA detection of AGEs, inflammatory factors and antioxidant indices detection

Serum levels of advanced glycation end products (AGEs), tumor necrosis factor-α (TNF-α), interleukin-6 (IL-6), interleukin-1β (IL-1β), superoxide dismutase (SOD), glutathione peroxidase (GSH-Px) and malondialdehyde (MDA) were determined using commercial ELISA kits (Youpin Biotechnology, China) according to the manufacturer’s protocols. The absorbance at 450 nm was measured by a Bio-Rad microplate reader (Bio-Rad, United States). Each sample was set with three replicate wells.

#### 2.2.4 Hematoxylin-eosin (HE) staining

Placental tissues were fixed in 4% paraformaldehyde solution (Biosharp, Anhui, China) for 24 h. After gradient dehydration, xylene transparency and paraffin embedding, continuous sections with a thickness of 5 μm were prepared using a Leica microtome. After dewaxing and rehydration, sections were stained with hematoxylin and eosin staining solution (Meilunbio, China). The morphological structure of placental tissues was observed under an Olympus optical microscope (Olympus, Japan).

#### 2.2.5 qRT-PCR for mRNA expression detection

Total RNA was extracted from placental tissues and HTR-8/SVneo cells using RNA extraction kits (Vazyme Biotech, Nanjing, China). RNA purity and integrity were verified before reverse transcription. cDNA synthesis and qRT-PCR amplification were performed using kits purchased from Vazyme Biotech (Nanjing, China). The detected genes included RAGE, IL1B, SERPINE1, NF-κB p65, Nrf2. Tubulin was used as the internal reference gene. The relative mRNA expression level was calculated by the 2^^−^ΔΔCt method.

#### 2.2.6 Western blot

Total protein was extracted from samples using RIPA lysis buffer containing protease and phosphatase inhibitors (Beyotime, Shanghai, China). Protein concentration was determined by BCA protein quantification kit (Beyotime, Shanghai, China). Equal amounts of protein were loaded for SDS-PAGE electrophoresis and transferred onto PVDF membranes (Millipore, United States). Membranes were blocked with 5% skimmed milk, and incubated with primary antibodies against RAGE, IL1B, SERPINE1, NF-κB p65, p-NF-κB p65, Nrf2, and Tubulin (1:1000, Immunoway, United States) at 4 °C overnight. After incubation with HRP-conjugated secondary antibody (1:5000, Immunoway, United States), protein bands were visualized using ECL luminescence reagent (Millipore, United States). ImageJ software was used to analyze the gray value of bands.

#### 2.2.7 Cell culture and intervention

Human placental trophoblast HTR-8/SVneo cells were cultured in RPMI-1640 medium (Gibco, United States) supplemented with 10% fetal bovine serum and 1% double antibiotics, and incubated at 37 °C with 5% CO_2_; five standardized cellular experimental groups were established for this study: CTRL group (5.5 mM normal glucose medium without any treatment as blank control), CTR + MAN group (5.5 mM glucose medium supplemented with mannitol at an osmolarity equivalent to 30 mM glucose), CTR + FPS group (5.5 mM normal glucose medium supplemented with 10 μM FPS-ZM1), HG group (30 mM high-glucose medium to mimic the *in vitro* GDM trophoblast injury microenvironment), HG + FPS group (30 mM high glucose supplemented with optimal concentration of RAGE selective inhibitor 10 μM FPS-ZM1), HG + QUE group (30 mM high glucose supplemented with 12.5 μM quercetin), HG + QUE + FPS group (co-treatment with 30 mM high glucose, 12.5 μM quercetin and 10 μM FPS-ZM1), FPS-ZM1 (Cat. No. HY-19370, MedChemExpress, United States; CAS No. 945714-67-0), a pharmacological inhibitor targeting RAGE-associated signaling, was dissolved in dimethyl sulfoxide (DMSO). The final concentration of DMSO was maintained consistently in all corresponding groups to exclude potential vehicle effects. Before intervention experiments, the cytotoxicity of FPS-ZM1 was evaluated using the Cell Counting Kit-8 (CCK-8) assay. HTR-8/SVneo cells were treated with different concentrations of FPS-ZM1 (0, 5, 10, 15, and 20 μM), and 10 μM was selected as the working concentration based on cell viability results. All cell intervention treatments lasted for 24 h before cell sample collection.

### 2.3 Statistical analysis

All data are expressed as mean ± standard deviation (x̄ ± s). GraphPad Prism 9.0 and R 4.3.0 were used for statistical analyses. For cellular experiments, each assay was performed in three independent biological replicates. Individual maternal rats served as biological replicates for *in-vivo* studies (n = 6-8 per experimental group). Multiple-group comparisons were performed using one-way ANOVA followed by Tukey’s *post hoc* multiple-comparison test. *P* < 0.05 was considered statistically significant.

## 3 Results

### 3.1 Network pharmacology analysis identifies AGE-RAGE signaling and predicts quercetin–RAGE interaction

Network pharmacology analysis was conducted to systematically explore the molecular basis of quercetin against GDM. After intersecting quercetin predicted targets, GDM pathogenic genes and differentially expressed placental genes from two independent GEO datasets, a total of 13 core hub genes were screened (Figures 1A–D). SERPINE1 was highlighted among top hub candidates due to its critical function in regulating placental vascular remodeling. These genes constructed a tightly correlated PPI network via the MCC algorithm (Figures 1E,F). KEGG enrichment analysis showed that the AGE-RAGE signaling pathway possessed the highest enrichment score, accompanied by oxidative stress and inflammatory response pathways (Figure 1G).

**FIGURE 1:**
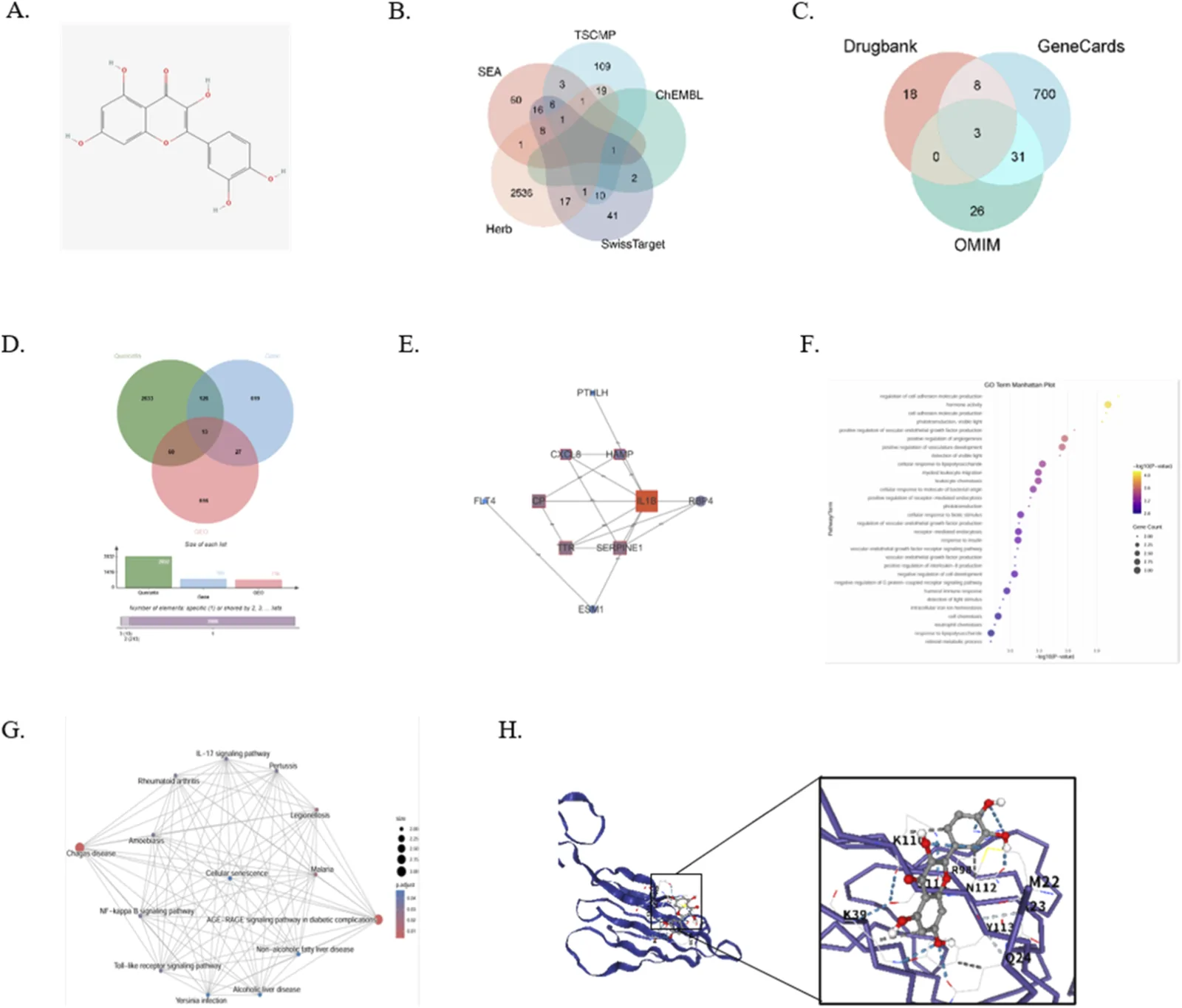
Network pharmacology analysis identifies AGE-RAGE signaling as a potential pathway of quercetin in GDM. **(A)** Chemical structure of quercetin. **(B)** Potential targets of quercetin predicted from TCMSP, ChEMBL, SwissTargetPrediction, SEA and Herb databases. **(C)** GDM-related genes screened from DrugBank, GeneCards and OMIM databases. **(D)** Venn diagram showing the intersection of quercetin targets, GDM-related genes and placental DEGs, resulting in 13 overlapping candidate genes. **(E)** Protein-protein interaction (PPI) network of the overlapping candidate genes. **(F)** GO functional enrichment analysis of the core genes. **(G)** KEGG pathway enrichment analysis of the core genes. **(H)** Molecular docking analysis showing the predicted binding mode and amino acid interactions between quercetin and RAGE.

To investigate the potential structural interaction between quercetin and RAGE, molecular docking analysis was performed using the human RAGE ligand-binding domain structure. Five candidate binding cavities were evaluated, and Cavity 5 was selected as the docking site based on structural characteristics and biological relevance. The selected pocket contained several potential interacting residues, including Met22, Lys39, Lys110, Ser111, Asn112, Tyr113, and Arg114. Docking analysis showed that quercetin could be accommodated within the predicted RAGE binding pocket and form multiple predicted interactions with surrounding amino acid residues (Figure 1H).

### 3.2 Molecular dynamics analysis of the predicted RAGE–quercetin complex

To further evaluate the stability of the selected RAGE–quercetin complex, a 100 ns molecular dynamics simulation was performed (Figures 2A–I). The RMSD and RMSF analyses showed that the complex remained relatively stable with moderate residue fluctuations (Figures 2A,B). The radius of gyration and SASA values remained consistent throughout the simulation, indicating preservation of the overall conformation (Figures 2C,D). Hydrogen bond analysis revealed persistent interactions between quercetin and RAGE, with Asn112 and Met22 identified as major interacting residues (Figures 2E,F). Furthermore, Gibbs free energy landscape and MM/PBSA analyses indicated a favorable energy state of the complex (Figures 2G–I). These results suggest that the predicted RAGE–quercetin complex exhibits dynamic stability during the simulation.

**FIGURE 2:**
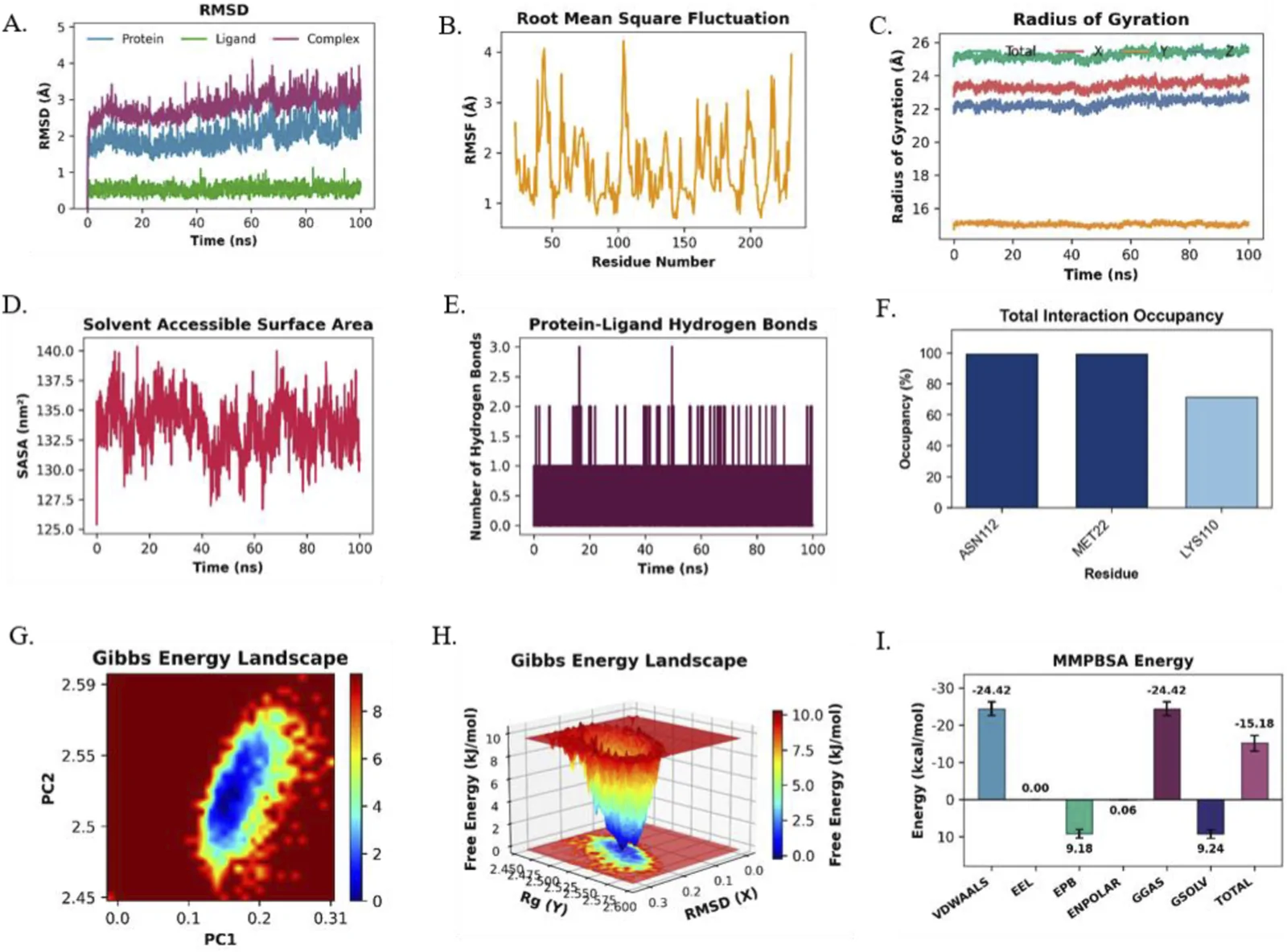
Molecular dynamics simulation analysis of the RAGE–quercetin complex. **(A)** RMSD analysis of the RAGE–quercetin complex during 100 ns molecular dynamics simulation. **(B)** RMSF analysis showing residue flexibility. **(C,D)** Radius of gyration and SASA analyses evaluating conformational stability. **(E,F)** Hydrogen bond analysis and residue interaction occupancy between RAGE and quercetin. **(G,H)** Gibbs free energy landscape analysis showing the low-energy conformational states. **(I)** MM/PBSA energy decomposition analysis of the RAGE–quercetin complex.

### 3.3 Quercetin improves metabolic disturbances and pregnancy-associated abnormalities in HFD/STZ-induced GDM rats

An HFD/STZ-induced GDM rat model was established to evaluate the protective effects of quercetin *in vivo*. Compared with control rats, GDM rats exhibited significantly increased fasting blood glucose levels, impaired glucose tolerance, and reduced insulin sensitivity, as indicated by elevated IPGTT-AUC and ITT-AUC values (Figures 3A,F–I). Quercetin treatment markedly improved glucose homeostasis and insulin responsiveness. Moreover, GDM rats showed decreased maternal weight gain, fetal weight, and placental weight at GD20, whereas quercetin intervention partially restored these pregnancy-associated abnormalities (Figures 3B–E). These results demonstrate that quercetin alleviates metabolic dysfunction and improves adverse pregnancy outcomes in GDM rats.

**FIGURE 3:**
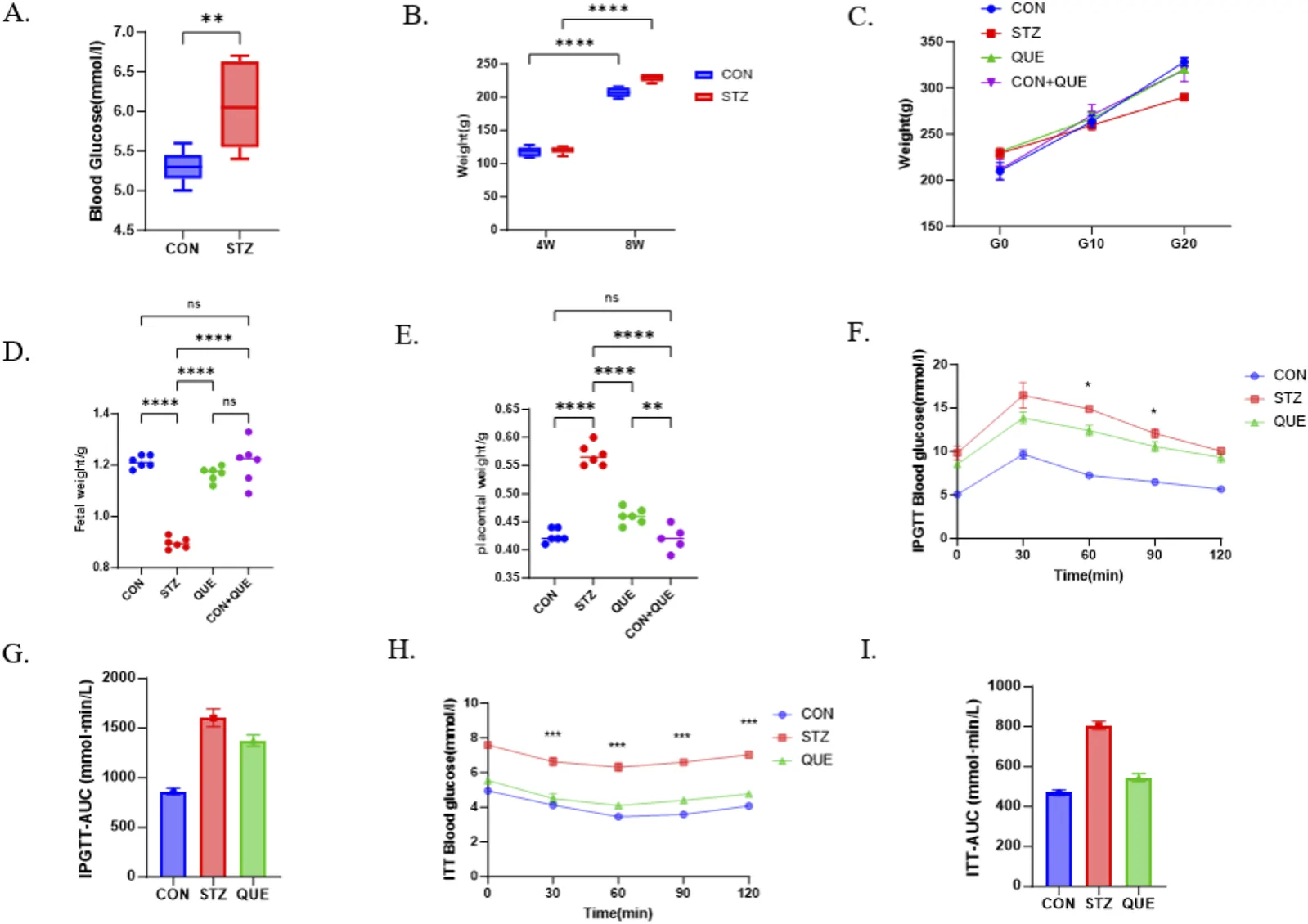
Quercetin ameliorates metabolic abnormalities and pregnancy-associated alterations in HFD/STZ-induced GDM rats. **(A)** Fasting blood glucose of rats after 4 weeks of high-fat diet feeding. **(B)** Body weight changes in rats during the 4-week high-fat diet feeding period (from 4 to 8 weeks of age). **(C)** Maternal body weight during gestation. **(D)** Body weight at GD20. **(E)** Placental weight at GD20. **(F)** Intraperitoneal glucose tolerance test (IPGTT) curves of each group. **(G)** Quantification of the area under the curve (AUC) for IPGTT. **(H)** Insulin tolerance test (ITT) curves of each group. **(I)** Quantification of the area under the curve (AUC) for ITT. Data are presented as mean ± SD, n = 6. *P < 0.05, **P < 0.01, ***P < 0.001, ****P < 0.0001.

### 3.4 Quercetin alleviates placental inflammation and oxidative stress in GDM rats

Placental pathological alterations were assessed by histological and biochemical analyses. HE staining revealed increased inflammatory cell infiltration and structural abnormalities in the placental labyrinth of GDM rats, which were attenuated after quercetin treatment (Figure 4A). Serum levels of pro-inflammatory cytokines, including TNF-α, IL-6, and IL-1β, were significantly elevated in GDM rats, while quercetin markedly reduced their concentrations (Figure 4B). GDM rats also exhibited increased oxidative stress, characterized by elevated MDA levels and decreased SOD and GSH-Px activities, whereas quercetin restored oxidative-antioxidant balance (Figure 4C). Consistently, placental mRNA and protein analyses showed that quercetin suppressed the enhanced expression of inflammatory mediators in GDM placentae (Figures 4D,E). Together, these findings indicate that quercetin protects against GDM-associated placental injury by attenuating inflammatory responses and oxidative stress.

**FIGURE 4:**
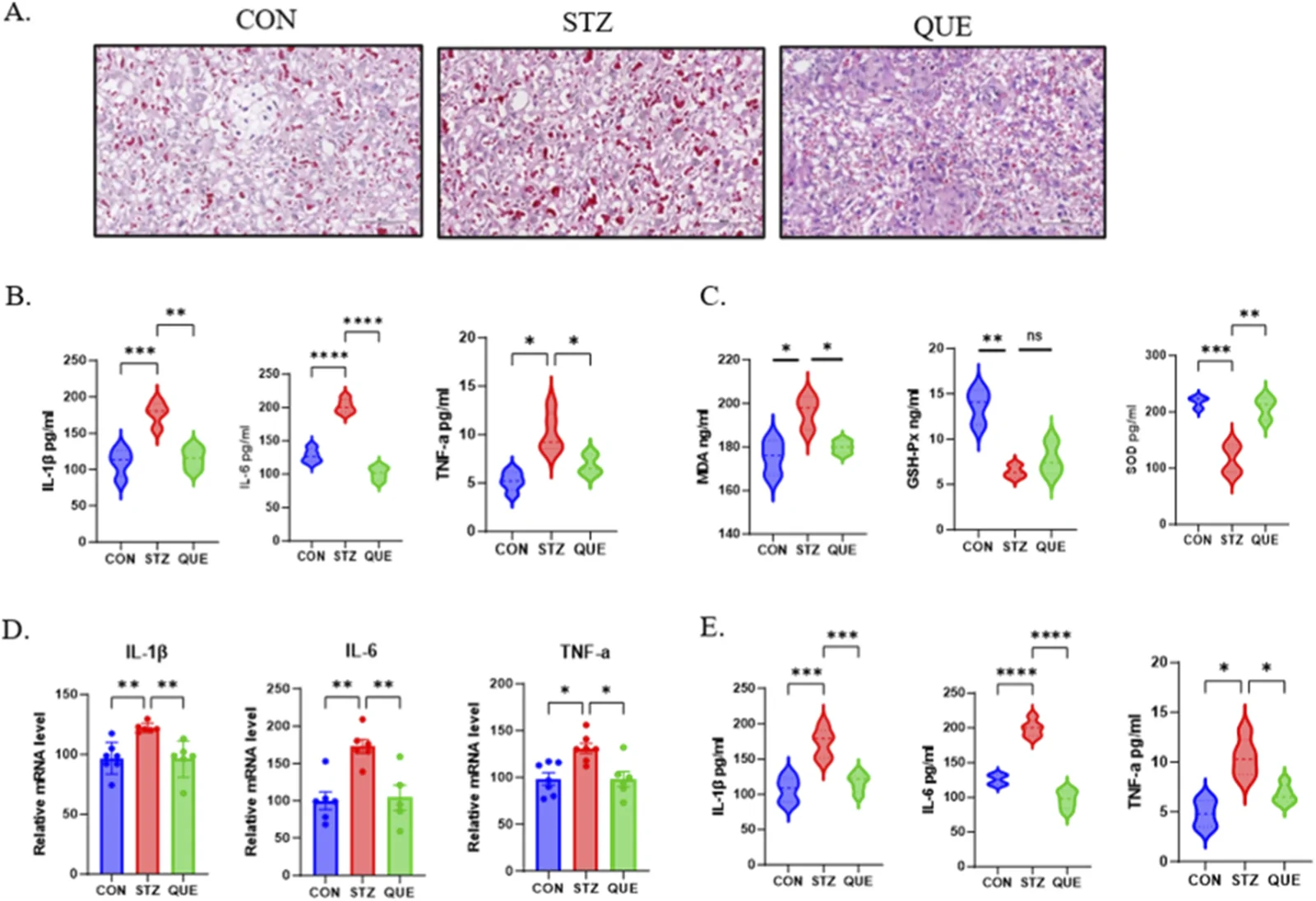
Quercetin alleviates placental histopathological damage, systemic inflammation, and oxidative stress. **(A)** Representative HE staining images of rat placental tissues (200× magnifica-tion). **(B)** Serum concentrations of TNF-α, IL-6 and IL-1β determined by ELISA. **(C)** Serum levels of MDA, SOD and GSH-Px for evaluation of oxidative stress status. **(D)** Relative mRNA expression of inflammatory factors (TNF-α, IL-6, IL-1β) in placental tissues detected by qRT-PCR. **(E)** Contents of inflammatory factors (TNF-α, IL-6, IL-1β) in placental tissue homogenate measured by ELISA. Data are presented as mean ± SD, n = 6. *P < 0.05, **P < 0.01, ***P < 0.001, ****P < 0.0001.

### 3.5 Quercetin modulates AGE-RAGE-associated signaling alterations in GDM placentae

Based on network pharmacology analysis, the AGE-RAGE signaling pathway was further investigated in placental tissues. Placental AGE accumulation and ROS production were significantly increased in GDM rats and were markedly reduced following quercetin administration (Figures 5A,B). qRT-PCR analysis showed that the expression levels of RAGE, SERPINE1, and NF-κB p65 were significantly elevated in GDM placentae, whereas Nrf2 expression was also increased, indicating an activated antioxidant response under oxidative stress conditions (Figures 5C–F). Western blot analysis further confirmed these changes at the protein level. Compared with control rats, GDM placentae exhibited increased expression of RAGE, phosphorylated NF-κB p65, SERPINE1, and Nrf2. Quercetin treatment significantly reduced the elevated levels of these pathway-related proteins, restoring their expression toward the control state (Figures 5G–L). These findings indicate that quercetin alleviates GDM-associated oxidative stress and inflammatory activation by modulating the AGE-RAGE-associated signaling network and normalizing stress-responsive Nrf2 activation in placental tissues.

**FIGURE 5:**
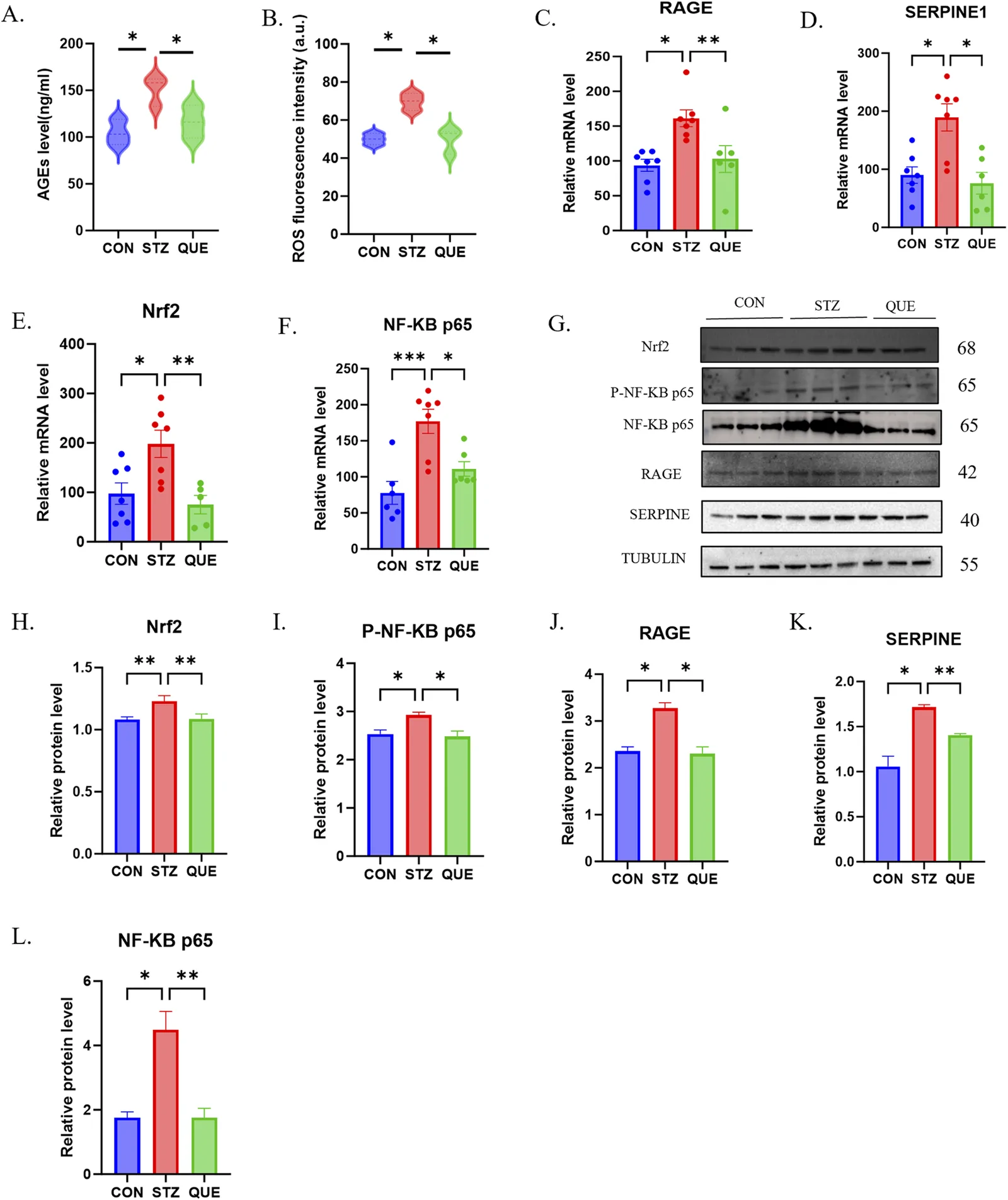
Quercetin modulates AGE-RAGE-associated oxidative and inflammatory signaling in placental tissues. **(A)** Placental AGEs levels measured by ELISA. **(B)** Relative ROS fluorescence intensity in placental tissues. **(C–F)** Relative mRNA expression of Nrf2, RAGE, SERPINE1, and NF-κB p65 detected by qRT-PCR. **(G–L)** Protein levels of Nrf2, RAGE, SERPINE1, total NF-κB p65, and p-NF-κB p65 detected by Western blot. Data are presented as mean ± SD, n = 6-8. *P < 0.05, **P < 0.01, ***P < 0.001, ****P < 0.0001.

### 3.6 Quercetin regulates AGE-RAGE-associated oxidative and inflammatory signaling in trophoblast cells

To investigate the protective effects of quercetin under high glucose conditions, HTR-8/SVneo cells were used as an *in vitro* trophoblast model. CCK-8 assays were performed to determine the appropriate concentrations of quercetin and FPS-ZM1. Quercetin showed no significant cytotoxicity at 10 μM, and FPS-ZM1 exhibited good cellular tolerance at 10 μM; therefore, these concentrations were selected for subsequent experiments (Figures 6A,B). High glucose exposure markedly increased intracellular ROS accumulation, whereas quercetin treatment significantly reduced ROS levels (Figure 6C). Moreover, high glucose stimulation increased the expression of RAGE, NF-κB p65, and SERPINE1, accompanied by stress-induced elevation of Nrf2 expression, while quercetin partially normalized these alterations (Figures 6D–G). Consistently, high glucose enhanced the secretion of inflammatory cytokines (IL-1β, IL-6, and TNF-α), whereas quercetin significantly attenuated their production (Figures 6H–J). Western blot analysis further confirmed that quercetin reversed high glucose-induced changes in RAGE, phosphorylated NF-κB p65, SERPINE1, and Nrf2 protein expression (Figures 6K–P).

**FIGURE 6:**
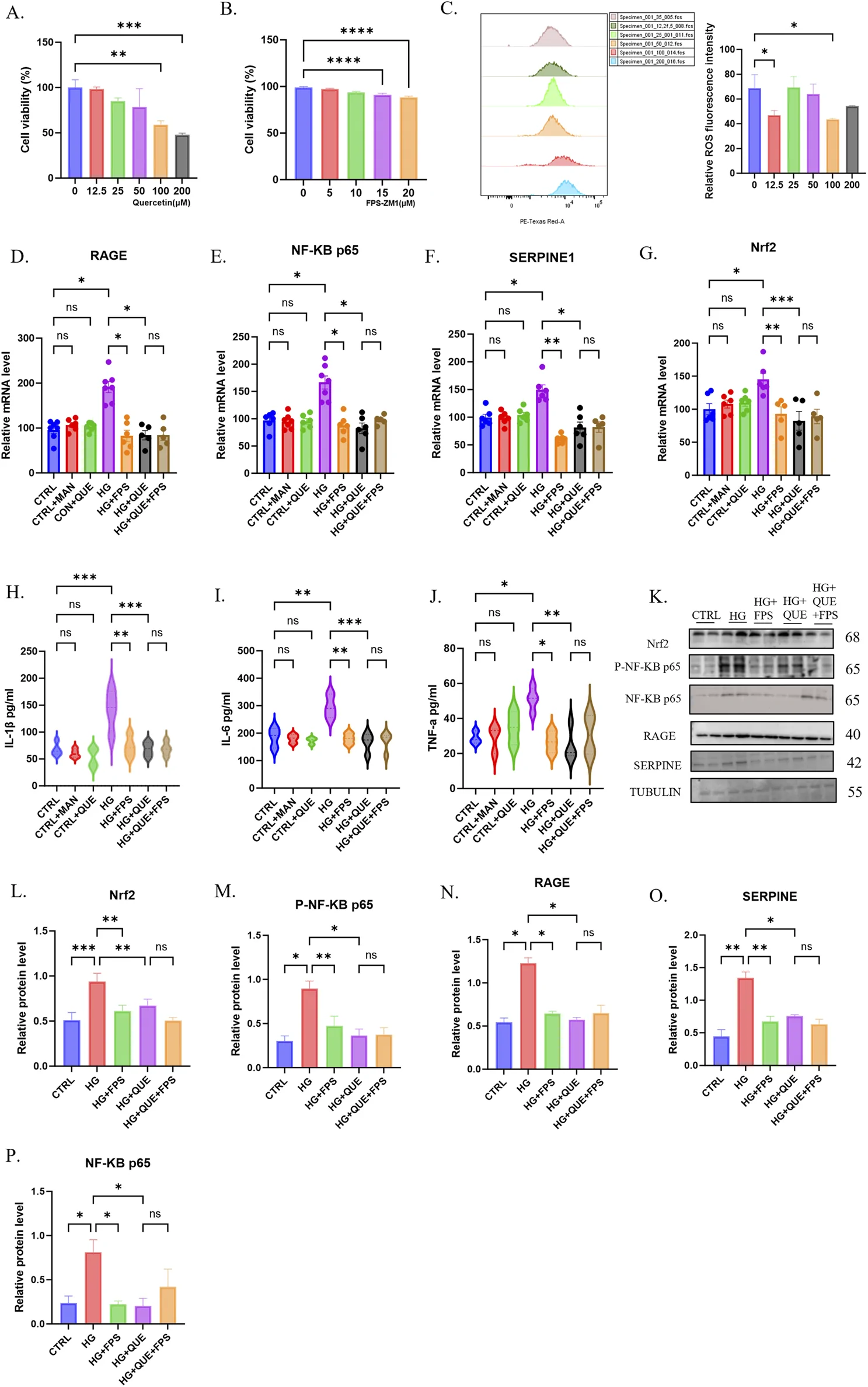
Quercetin attenuates high glucose-induced trophoblast injury through AGE-RAGE-associated signaling. **(A,B)** Cell viability of HTR-8/SVneo cells treated with quercetin or FPS-ZM1 evaluated by CCK-8 assay. **(C)** Intracellular ROS fluorescence intensity detected by flow cytometry. **(D–G)** Relative mRNA expression levels of RAGE, NF-κB p65, SERPINE1 and NRF2 measured by qRT-PCR. **(H–J)** Levels of IL-1β, IL-6 and TNF-α in culture supernatants determined by ELISA. **(K)** Representative Western blot images of NRF2, phosphorylated NF-κB p65, total NF-κB p65, RAGE and SERPINE1. **(L–P)** Quantification of relative protein levels. Data are presented as mean ± SD, n = 6. *P < 0.05, **P < 0.01, ***P < 0.001.

To further examine the involvement of RAGE signaling, FPS-ZM1 was applied as a pharmacological inhibitor. No significant phenotypic difference was observed between the HG + QUE group and the HG + QUE + FPS-ZM1 group regarding ROS generation, inflammatory cytokine secretion, and AGE-RAGE-related molecular markers (Figures 6C–P). Together, these cellular data demonstrate that quercetin mitigated high-glucose-triggered trophoblast oxidative stress and inflammatory activation, accompanied by modulation of AGE-RAGE-related molecular profiles.

## 4 Discussion

Gestational diabetes mellitus (GDM)-associated placental dysfunction is characterized by complex alterations in metabolic regulation, oxidative balance, and inflammatory responses. In the present study, we demonstrated that quercetin alleviated placental injury under diabetic conditions and improved the abnormal oxidative-inflammatory state associated with GDM. By integrating network pharmacology, molecular docking, molecular dynamics simulation, animal experiments, and trophoblast cell validation, our findings suggest that quercetin exerts placental protective effects through coordinated regulation of AGE-RAGE-associated oxidative and inflammatory signaling.

Quercetin is a naturally occurring flavonoid with well-established antioxidant, anti-inflammatory, and metabolic regulatory properties. Previous studies have demonstrated that quercetin can improve diabetic complications through regulation of oxidative stress, inflammatory responses, and glucose metabolism, supporting its potential as a multi-target bioactive compound (Boots et al., 2008; Eid and Haddad, 2017; Anand David et al., 2016). However, the molecular mechanisms underlying its protective effects in GDM-associated placental injury remain incompletely understood. In the present study, network pharmacology analysis identified AGE-RAGE-associated signaling as a potential pathway related to quercetin activity, which was further supported by molecular simulation and experimental validation. The AGE-RAGE axis is an important metabolic stress-responsive pathway in diabetic conditions, and its activation contributes to ROS accumulation and inflammatory signaling activation (Coskun et al., 2005; Mahmoud et al., 2013; Peng et al., 2017). Therefore, modulation of AGE-RAGE-associated signaling may represent an important mechanism through which quercetin counteracts hyperglycemia-induced placental injury.

The protective effects of quercetin may be closely associated with regulation of the oxidative-inflammatory interaction induced by metabolic stress. AGE-RAGE activation promotes ROS generation, which subsequently enhances NF-κB signaling and inflammatory cytokine production, including TNF-α, IL-1β, and IL-6 (Peng et al., 2017; Ramasamy et al., 2011; Goldin et al., 2006). This process forms an important mechanism through which metabolic abnormalities are translated into persistent inflammatory injury. Previous studies have shown that quercetin can attenuate oxidative stress and inflammatory activation through regulation of ROS-associated pathways and NF-κB signaling (Lawrence, 2009; Bryan et al., 2013). Consistent with these findings, the present study suggests that quercetin may reduce placental injury by alleviating excessive oxidative and inflammatory responses associated with GDM.

In addition to reducing oxidative stress, quercetin may influence endogenous antioxidant adaptation under diabetic conditions. It should be noted that Nrf2 elevation observed in GDM placentae represents a compensatory adaptive response triggered by sustained oxidative burden, rather than a fully effective antioxidant output. Under persistent hyperglycemic stress, accumulated ROS drive Nrf2 upregulation; however, this compensatory response is insufficient to reverse oxidative injury, as reflected by concurrently elevated MDA and pro-inflammatory mediators. Upon quercetin intervention, the overall oxidative load is mitigated, relieving the stress-driven compensatory induction of Nrf2. Further assays measuring nuclear-translocated Nrf2 and downstream effector proteins such as HO-1 or NQO1 would provide deeper mechanistic insight into Nrf2-mediated antioxidant responses, which constitutes one limitation of the present study. Nrf2 is a central regulator of cellular antioxidant defense and responds to increased oxidative burden by activating antioxidant response pathways. Previous studies have suggested that enhanced Nrf2 expression in diabetic conditions may represent a compensatory response attempting to maintain redox homeostasis under excessive oxidative stress (Sies et al., 2017; Juhan-Vague et al., 2003). However, persistent metabolic stress may result in continuous ROS production, leading to activation of antioxidant pathways that are insufficient to completely restore normal redox balance. Therefore, alterations in Nrf2 expression should be considered as a reflection of cellular adaptation to oxidative challenges. In the present study, changes in Nrf2 expression following GDM induction and quercetin treatment suggest that quercetin may alleviate oxidative stress burden and subsequently influence antioxidant adaptation responses.

SERPINE1 represents another potential molecular component involved in quercetin-mediated improvement of placental dysfunction. SERPINE1 encodes plasminogen activator inhibitor-1 (PAI-1), which participates in extracellular matrix remodeling, vascular regulation, and inflammatory responses. Increased SERPINE1 expression has been associated with insulin resistance, metabolic syndrome, and diabetic vascular complications (Loskutoff and Samad, 1998; Deane et al., 2012). Because SERPINE1 is regulated by multiple metabolic and inflammatory signals, its alteration may reflect broader changes in the cellular stress response during diabetic injury. The reduction of SERPINE1 expression following quercetin treatment observed in this study suggests that regulation of SERPINE1-associated processes may contribute to the restoration of placental homeostasis.

To further explore the involvement of RAGE-associated signaling, FPS-ZM1 was applied as a pharmacological inhibitor of RAGE. Previous studies have demonstrated that FPS-ZM1 can reduce AGE-RAGE-mediated oxidative stress and inflammatory responses in diabetic injury models, supporting its use for investigating RAGE-associated mechanisms (Deane et al., 2012; Hudson and Lippman, 2018). Interestingly, combined treatment with FPS-ZM1 and quercetin did not yield additional significant improvement compared with FPS-ZM1 alone. A plausible interpretation is that pharmacological blockade of RAGE by FPS-ZM1 already blunts major AGE-RAGE-dependent oxidative-inflammatory cascades, whereby the RAGE-dependent component of quercetin’s protective effect is largely occluded. As a multi-target flavonoid, quercetin exerts broad protective actions including direct radical-scavenging antioxidant effects. Nevertheless, no further phenotypic benefit was detected upon co-treatment once RAGE signaling was suppressed. We cannot fully exclude potential off-target effects of FPS-ZM1, which is an inherent caveat of pharmacological perturbation. Genetic-based RAGE manipulation will help to further validate these observations in future work.

Compared with previous studies focusing mainly on the general antioxidant and anti-inflammatory effects of quercetin, the present study provides a comprehensive mechanistic investigation integrating computational prediction, animal models, cellular validation, and pathway intervention. Network pharmacology has been increasingly applied to investigate the complex biological actions of natural compounds by identifying potential targets and signaling networks (Hopkins, 2008; Li and Zhang, 2013). Molecular docking and molecular dynamics simulation further provide structural insights into potential compound-target interactions and molecular stability (Trott and Olson, 2010; Karplus and McCammon, 2002; Hollingsworth and Dror, 2018). Through this integrated approach, our findings extend current understanding by suggesting that quercetin may regulate a coordinated signaling network involving AGE-RAGE-associated responses, oxidative stress adaptation, inflammatory activation, and SERPINE1 regulation during GDM-associated placental injury.

Several limitations should be acknowledged. First, although the present study provides multiple lines of evidence supporting the potential involvement of AGE-RAGE-associated signaling, direct physical molecular interaction between quercetin and RAGE requires further experimental biochemical confirmation. Second, conclusions drawn from FPS-ZM1 pharmacological intervention remain inconclusive based on our cellular assays. Future genetic-based RAGE manipulation will help to definitively clarify the functional contribution of RAGE signaling in this experimental context. Third, validation using human placental samples and primary trophoblast cell models will be important to improve the clinical translational relevance of our findings. In addition, while normoglycemic Control + QUE animals were included and maternal phenotypic outcomes including maternal weight and fasting blood-glucose have been reported in this manuscript, our analyses were primarily focused on pathological GDM-related placental injury. Therefore, placental molecular profiling of quercetin-treated normal pregnancy was beyond the scope of the present investigation and awaits future dedicated studies. Finally, the poor *in-vivo* bioavailability of quercetin represents a substantial barrier hindering future clinical translational applications.

## 5 Conclusion

Quercetin alleviated GDM-associated metabolic disturbance, placental injury, and adverse pregnancy outcomes in HFD-STZ-induced rats. Computational simulations predict a potential interaction between quercetin and the ligand-binding domain of RAGE. Quercetin modulated the AGE-RAGE-associated oxidative-inflammatory signaling network, restored maternal-fetal redox-inflammatory homeostasis, normalized stress-provoked Nrf2 adaptive responses, and down-regulated SERPINE1 expression. These findings support quercetin as a candidate agent for ameliorating GDM-related placental damage via suppression of AGE-RAGE-driven oxidative and inflammatory responses (Figure 7).

**FIGURE 7:**
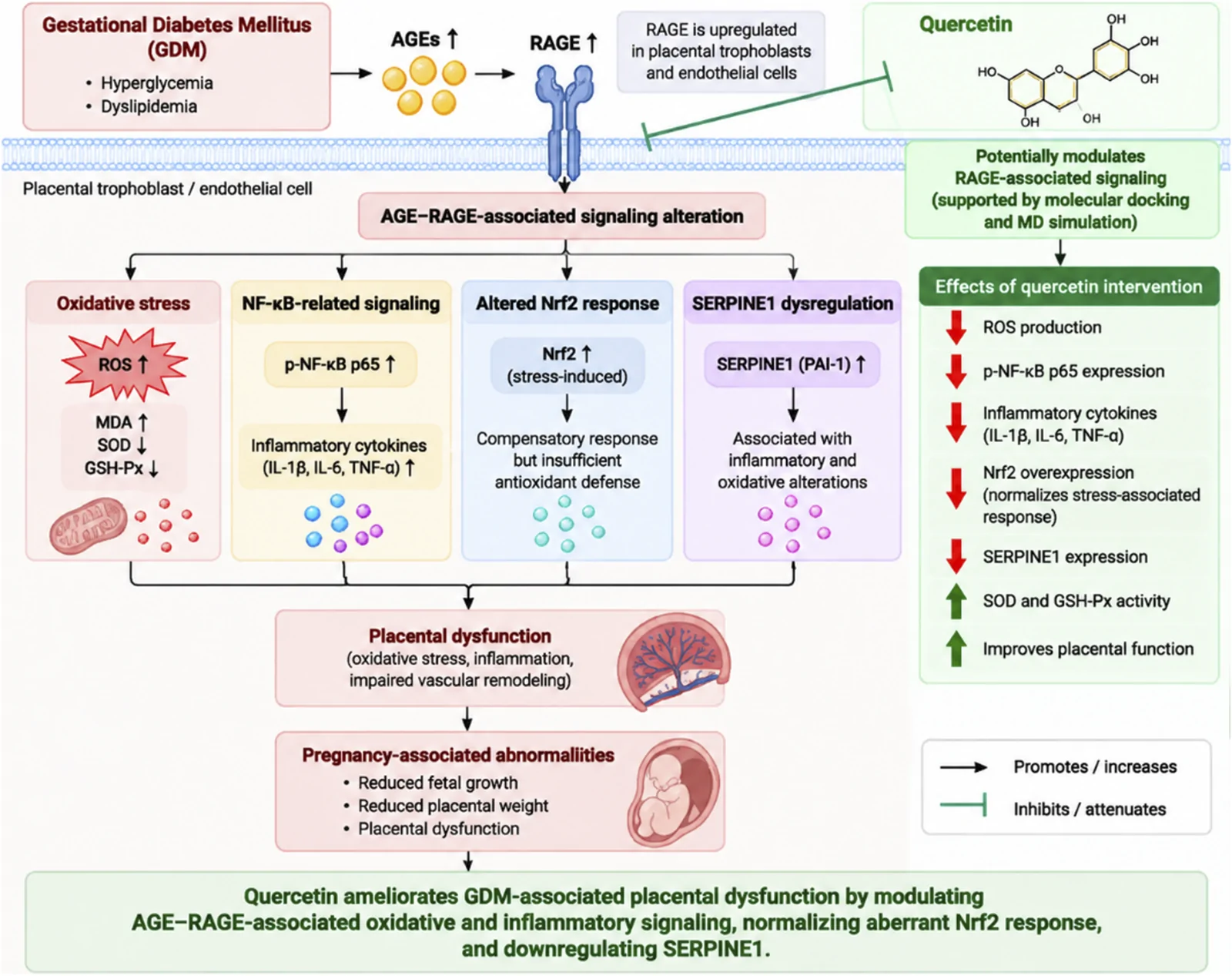
Proposed molecular mechanism underlying the protective effects of quercetin against GDM-associated placental dysfunction. This schematic illustrates the proposed mechanism by which quercetin alleviates placental dysfunction in GDM through modulation of AGE-RAGE-associated oxidative and inflammatory signaling. Hyperglycemic conditions promote AGE accumulation and RAGE-associated signaling alterations, contributing to excessive ROS generation and inflammatory responses mediated by NF-κB-related pathways. Concurrently, oxidative stress induces an adaptive Nrf2 response, which is accompanied by dysregulated SERPINE1 expression and impaired placental function. Quercetin intervention attenuates oxidative stress, inflammatory signaling alterations, and SERPINE1 dysregulation, thereby improving GDM-associated placental abnormalities.

## Data Availability

The human placental transcriptome dataset GSE263483 analyzed in this work was downloaded from the Gene Expression Omnibus public database. All raw experimental datasets including unprocessed Western blot images, original flow cytometry files and bioinformatic analysis scripts generated during this research are available from the corresponding authors upon reasonable request.
